# Mixed Vaginal Infections and Their Predictors Among Women With Abnormal Vaginal Discharges Attending Gynecological Clinics in Western Uganda: A Cross-Sectional Study

**DOI:** 10.1155/ipid/6511013

**Published:** 2025-08-28

**Authors:** Salma Khamis Said, Marie Pascaline Sabine Ishimwe, Musa Kasujja, Peter Okello, Khadija Khamis Said, Maxwell Okello, Emmanuel Okurut, Theoneste Hakizimana

**Affiliations:** ^1^Department of Obstetrics and Gynecology, Kampala International University, Ishaka, Uganda; ^2^Department of Pediatrics and Child Health, Kampala International University, Ishaka, Uganda; ^3^Department of Pediatrics and Child Health, Lumumba District Hospital, Lumumba, Zanzibar, Tanzania

**Keywords:** abnormal vaginal discharges, gynecological clinic, mixed vaginal infections, predictors, western Uganda

## Abstract

**Background:** Mixed vaginal infection involves the simultaneous presence of at least two types of vaginitis, including bacterial vaginosis (BV) and vulvovaginal candidiasis (VVC), BV and *Trichomonas vaginalis* (TV), or TV and VVC. This condition disrupts the vaginal milieu, resulting in significant diagnostic and therapeutic challenges, recurrent infections, and increased antimicrobial resistance. This study aimed to assess the mixed vaginal infections and their predictors among women with abnormal vaginal discharges attending gynecological clinics in western Uganda.

**Methods:** A cross-sectional study was conducted with 146 participants from the gynecology clinic at Fort Portal Regional Referral Hospital (FRRH) from January 2024 to April 2024. Data collection included medical record reviews, structured interviews, and swab culture testing. Data were compiled in Microsoft Excel 16.0, cleaned, and imported into SPSS Version 22.0 for analysis. Logistic regression and descriptive statistics were utilized for data analysis.

**Results:** The overall prevalence of mixed vaginal infections among women with abnormal vaginal discharges attending the gynecological clinic at FRRH was 28.1% (41/146). Among those with mixed infections, the most common type was BV/VVC (19.2%), followed by TV/BV (4.8%) and TV/VVC (4.1%). Significant factors associated with mixed vaginal infections were rural residence (adjusted odds ratio [aOR] = 2.9, 95% confidence interval [CI]: 1.1–7.5, *p* = 0.03), HIV-positive status (aOR = 4.5, 95% CI: 1.4–14.3, *p* = 0.01), multiple sexual partners (aOR = 5.5, 95% CI: 1.31–23.8, *p* = 0.02), vaginal douching (aOR = 4.6, 95% CI: 1.6–13.3, *p* < 0.001), and having two or more previous vaginal infections (aOR = 9.5, 95% CI: 2.2–41.1, *p* = 0.001).

**Conclusions:** A high prevalence of mixed vaginal infections was observed among women at FRRH, with BV/VVC being the most frequent combination among those with mixed infections. Identified risk factors included rural residence, HIV-positive status, multiple previous infections, multiple sexual partners, and vaginal douching. These results emphasize the need for comprehensive diagnostic, therapeutic, and preventive strategies to manage mixed vaginal infections effectively.

## 1. Introduction

Vaginal infections are common gynecological conditions that significantly affect women's health worldwide. The most prevalent types of vaginitis include bacterial vaginosis (BV), vulvovaginal candidiasis (VVC), and *Trichomonas vaginalis* (TV). These infections can occur individually or concurrently, leading to mixed vaginal infections where at least two types of vaginitis are present simultaneously. Mixed vaginal infections disrupt the vaginal milieu, complicating diagnosis and treatment, and often result in recurrent infections and increased antimicrobial resistance [1, 2]. Globally, the prevalence of mixed vaginal infections varies widely, with reports ranging from 4.44% to 59.1% [3, 4]. These infections pose significant public health challenges, particularly in low-income settings where diagnostic facilities are limited and empirical treatment is common [5].

In Africa, Yalew et al. found an 8.3% prevalence of mixed vaginal infections among pregnant Ethiopian women, and in Egypt, Shawaky et al. reported a higher rate of 24% among this group, with attributes to the various techniques, including molecular methods, used by Shawaky and colleagues to identify different species involved in vaginitis [6, 7]. In Cameroon the study was conducted by Kamga in pregnant women; they found that the prevalence of mixed vaginal infection in their study was 9.1% [8].

In Uganda, the burden of mixed vaginal infections remains poorly characterized, highlighting the need for data to inform effective clinical management and public health strategies [9]. Existing literature highlights the complexity of diagnosing and managing mixed vaginal infections. Factors such as rural residence, HIV-positive status, multiple sexual partners, and vaginal douching have been identified as significant risk factors [6, 8, 10]. Few studies conducted in Uganda have primarily examined single infections, with BV identified as the most common pathogen, with a prevalence of 57% [11], while *Trichomonas vaginitis* was found to be the least frequently detected pathogen, at a prevalence of 9% [9].

However, there is still a paucity of data on the prevalence and associated factors of mixed vaginal infections among women presenting with abnormal vaginal discharge in Uganda.

Addressing mixed vaginal infections is crucial; by improving the diagnosis, treatment, and prevention of mixed vaginal infections, we can enhance women's reproductive health, reduce morbidity, and empower women to lead healthier lives [12].

This study aimed to assess the mixed vaginal infections and its predictors among women attending the gynecology clinics in western Uganda. By identifying the most common types of mixed infections and their associated risk factors, this study will contribute to improving diagnostic, therapeutic, and preventive strategies for mixed vaginal infections.

## 2. Materials and Methods

### 2.1. Study Design and Setting

A cross-sectional study was conducted in the gynecology department of Fort Portal Regional Referral Hospital (FRRH) between January 2024 and April 2024. It serves as a government hospital and a satellite teaching hospital for Kampala International University (KIU) in Uganda, and it is well established with a bed capacity of about 333 beds, serving a population of around 5 million people, including one city, seven districts, and parts of the Eastern Democratic Republic of Congo. FRRH is approximately 294 km from Kampala. The gynecology clinic at FRRH sees an average of 20–30 patients per week, amounting to about 1440 visits annually. The hospital is well-equipped with laboratory services, including culture and sensitivity testing, managed by a laboratory technician.

### 2.2. Sample Size Calculation

The sample size was determined based on Kish and Leslie's formula (1965).

(1) $$\begin{array}{c} n=\frac{Z^{2}p\left(1-p\right)}{d^{2}}, \end{array}$$

whereby,

*n*: estimated minimum sample size required.

*p* = proportion of characteristics in a sample (37.1%) in Nigeria [13].

*Z* = 1.96 (for 95% confidence interval).

*d* = Margin of error set at 5%

(2) $$\begin{array}{c} n=\frac{{\left(1.96\right)}^{2}\times 0.371\;\left(1-0.0371\right)}{{\left(0.05\right)}^{2}}=358. \end{array}$$

Therefore, the sample size required for objective one will be = 358.

Adjusting the sample size for a finite population, according to hospital records, FRRH recorded an average of 212 cases of abnormal vaginal discharge between 1st April and June 1st, 2023, which matched the intended data collection period. Consequently, the study population for this period consisted of 212 cases.

(3) $$\begin{array}{c} \text{Sample size }\left(N\right)=\frac{n_{s}}{1+\left(\left(n_{s}-1\right)/n\right)}, \end{array}$$

where *N*: adjusted population size; *n*_*s*_: estimated sample size and *n*: population under study *N* = 358/(1 +  (358 – 1)/212) = 133 participants.

Adding 10% for nonrespondents, the required sample size will be 146 participants.

### 2.3. Eligibility Criteria

#### 2.3.1. Inclusion Criteria

All nonpregnant women aged between 15 and 61 years and presenting with features of vaginal infection.

#### 2.3.2. Exclusion Criteria

Women experiencing heavy vaginal bleeding or those who had been on antibiotic therapy within the 2 weeks prior to the interview were excluded from the study.

#### 2.3.3. Data Collection Instruments

A checklist was used to collect information from patients who consented. One part of the checklist gathered data on sociodemographic and clinical factors, while another part collected information from swab samples, including the types of mixed microorganisms isolated. The questionnaire was translated into Rutooro for participants who could not understand English.

#### 2.3.4. Study Procedure

Patients presenting with symptoms of vaginitis, such as vaginal pruritus (itching) and abnormal vaginal discharge, at the gynecology clinic of FRRH were informed about the study and provided with written consent. Their primary complaints were recorded, followed by a detailed history to assess the symptoms, the duration since onset, and other potential predictive factors for mixed vaginitis.

During the examination, the characteristics of abnormal vaginal discharge and other signs of vaginitis, such as vaginal soreness and excoriation, were recorded with the patient in a supine position. For microbial isolation, a high vaginal swab was taken by inserting a sterile vaginal speculum to visualize the posterior fornix. A sterile cotton swab was then inserted into the posterior fornix and gently rotated to collect the sample. The swab was placed back into its tube, labeled with the patient's study number, initials, and date, and transported in a cool box to the microbiology laboratory at FRRH for analysis. The patient received treatment according to Uganda's clinical guidelines, and the researcher followed up on the laboratory results.

### 2.4. Sample Collection, Processing, and Analysis

#### 2.4.1. Specimen Collection

A high vaginal swab was collected using a sterile swab stick and speculum, inserted and rotated to gather vaginal discharge. The swab was immediately returned to its case to avoid contamination and transported to the laboratory within 30 min for examination and inoculation.

#### 2.4.2. Sample Processing

The swab sample was mixed with normal saline on a glass slide and examined under a microscope, revealing motile parasites (*Trichomonas vaginalis*), pus cells, yeast-like cells, and a motile bacterial background, with clue cells also observed. A smear of the sample was prepared, air-dried, heat-fixed, and Gram stained using crystal violet, Lugol's iodine, acetone-alcohol, and neutral red to detect clue cells indicating bacterial infection. Samples were inoculated on blood agar, chocolate agar, MacConkey's agar, and Sabouraud dextrose agar. Chocolate agar plates were incubated with 5%–10% carbon dioxide, blood agar assessed hemolysis types, Sabouraud dextrose agar promoted *Candida* growth, and MacConkey's agar supported Gram-negative bacteria growth. All plates were incubated at 37°C for 18–24 h.

#### 2.4.3. Colonies Appearance

After incubation, colonies on blood agar showed beta-hemolysis. MacConkey's agar revealed large pink, moist, sticky colonies typical of lactose-fermenting Gram-negative rods. *Candida* growth on Sabouraud dextrose agar indicated VVC.

#### 2.4.4. Diagnosis of BV

BV was diagnosed according to Amsel criteria: the presence of ≥ 3 of 4 features—(i) homogeneous grey-white discharge; (ii) vaginal pH > 4.5; (iii) positive whiff test with 10% KOH; and (iv) clue cells constituting > 20% of epithelial cells on Gram-stained smear [14–16].

#### 2.4.5. Study Variables

The study assessed various independent variables to understand their association with mixed vaginal infections among women. Demographic characteristics such as age were categorized into groups (< 20, 20–29, 30–39, and ≥ 40), and residence was classified as urban or rural. Clinical history variables included HIV status, determined through medical records, previous history of vaginal infections (none, one, or two or more), and the duration of symptoms from onset, all gathered via patient interviews. Behavioral factors included the number of sexual partners within the past year (one, two, or more) and whether the participant engaged in vaginal douching, both recorded through direct questioning.

The primary dependent variable was the presence of mixed vaginal infections, defined as the simultaneous occurrence of at least two types of vaginitis (BV, VVC, TV) confirmed through laboratory analysis. Additional variables such as cigarette smoking and diabetes were recorded as yes or no through patient interviews and medical history, respectively. History of abortions and menopause status (premenopause, menopause, or postmenopause) were also assessed via patient interviews and medical history.

### 2.5. Data Quality Control

To ensure data reliability and validity, several quality control measures were implemented. Personnel received comprehensive training on study protocols, and Standard Operating Procedures (SOPs) were strictly followed to maintain consistency and accuracy. Sterile techniques were used for specimen collection, and samples were transported to the laboratory within 30 min to preserve their integrity.

In the laboratory, high-quality reagents and calibrated equipment were used, with results double-checked by independent technicians to ensure accuracy. Data was entered into a secure, password-protected electronic database to minimize errors, and random samples were cross-verified with original documents. Periodic monitoring and regular internal audits ensured adherence to protocols and continuous improvement of study processes.

### 2.6. Data Analysis

Data collected from the questionnaires were entered into Microsoft Excel 2016 and subsequently exported to SPSS Version 22.0 for analysis. Continuous variables, such as age, were summarized using means, medians, standard deviations, and interquartile ranges. Categorical data, like education level, were analyzed to calculate proportions, percentages, and frequencies.

For Objective 1, the prevalence of mixed vaginal infections among women with abnormal vaginal discharge attending the gynecologic clinic at FRRH was determined. This was calculated as the percentage of patients diagnosed with mixed vaginal infection out of all patients attending the gynecologic outpatient clinic. Different patterns of mixed vaginal infection were summarized as percentages and frequencies and depicted using a pie chart.

For Objective 2, factors associated with mixed vaginal infections were assessed using binary logistic regression. Variables with a *p*-value of less than or equal to 0.2 in the univariate analysis were considered for multivariate analysis. A significance level of 5% was used, with a *p*-value of less than or equal to 0.05 indicating a statistically significant association between the variables. The results were presented in pie charts and tables to facilitate effective interpretation and discussion.

## 3. Results

### 3.1. Characteristics of the Study Participants

In this study 146 women were recruited from the gynecology clinic of FRRH with a response rate of 100%. The mean age of study participants was 28.1 years (SD = 2.17). The majority were in the age groups of 20–29 years and 30–39 years, each group representing 51 (34.93%) of the total sample.

The majority were from urban areas, 77 (52.7%); married, 83 (56.9%); attended primary school, 52 (35.6%); and were unemployed, 90 (61.6%) (Table 1).

**Figure 1:**
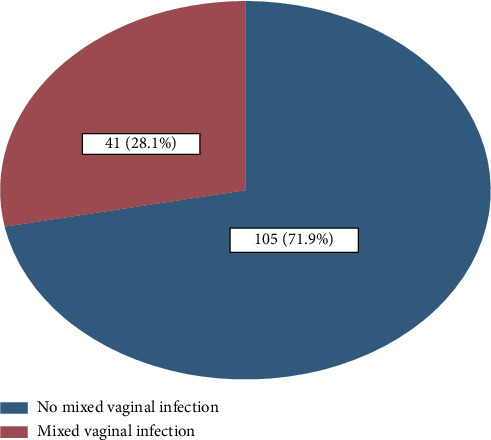
Prevalence of mixed vaginal infections among women with abnormal vaginal discharges attending the gynecology clinic at FRRH (*N* = 146).

Of 146 women, 41/146 were having mixed vaginal infections, translating to the prevalence of 28.1% (95% CI: 21.3–36.0), with 19.2% (28/146) having VVC + BV, 4.8% (7/146) having TV+ BV, and 4.1% (6/146) having VVC + TV (Figure 1).

To identify factors associated with mixed vaginal infections, univariate analysis carried out showed that age < 20, residence in rural areas, cigarette smoking, positive HIV serostatus, history of previous vaginal infections, number of sexual partners ≥ 2, practice of vaginal douching, previous history of abortions, and being in menopause were significant. After removing confounders at the multivariate level of analysis, residence in rural areas, positive HIV serostatus, history of previous vaginal infections, number of sexual partners ≥ 2, and practice of vaginal douching remained independently significant. Precisely, rural residents were 2.9 times more likely to develop mixed infections compared to urban residents. HIV-positive women had a 4.5-fold higher risk, and those with multiple sexual partners had a fivefold increase in risk. Vaginal douching increased the likelihood by 4.6 times. Additionally, women with a history of two or more vaginal infections in the past year were 9.5 times more likely to have mixed infections, highlighting the substantial impact of previous infections on current health risks (Table 2).

## 4. Discussion

In this study, the prevalence of mixed vaginal infections among women attending the gynecological ward at FRRH was found to be 28.1%. This rate aligns with global reports, which indicate a prevalence range of 5%–30% [1].

Shawaky et al. reported a lower prevalence of 24% among women visiting gynecology departments in Egypt [6]. This discrepancy may be due to the inclusion of all women in their study, while our study focused solely on women with abnormal vaginal discharges. Additionally, variations in healthcare infrastructure, public health practices, and potentially less sensitive diagnostic techniques could contribute to this difference. A prospective multicenter clinical study in the United States using Nucleic Acid Amplification Tests (NAATs) found a lower prevalence of 25% [17]. The difference could be attributed to the clinical study setting and its design. More advanced diagnostic techniques, such as NAATs, can detect infections more accurately, leading to higher reported prevalence rates, but better access to healthcare and education in the United States compared to Uganda may explain the higher prevalence in FRRH. In North India, Kalia et al. found a 20.5% prevalence of mixed infections [18], which is lower than our finding. This can be explained by the slight difference in the study population; their study only considered co-infections of BV and VVC among all married women, regardless of symptoms. In contrast, our study focused solely on women with vaginal discharge, which may indicate a higher burden of infection. Bhargava et al. reported an 18.1% prevalence of mixed infections in Nepal [5]; however, they also considered only BV and VVC co-infections, which can explain this disparity. In Uganda, Mujuzi et al. found a prevalence of 17.5% for mixed infections [19]. Their study included all women seeking reproductive health services, whether pregnant or not, and did not specifically focus on those with symptoms of vaginitis, such as vaginal discharge. The inclusion of asymptomatic women and those without specific symptoms of vaginitis can lead to a lower reported prevalence.

On the other hand, Donders and colleagues reported a prevalence of 35% in China [20], which is higher than our finding. They used molecular techniques, which are more sensitive and specific than the methods used in our study, potentially explaining the higher prevalence. More advanced diagnostic techniques can detect mixed infections more accurately, leading to higher reported prevalence rates [21]. In Nigeria, Oparaugo et al. found a 37.6% prevalence of mixed vaginal infections in a longer study conducted in Lagos [13]. This higher rate could be due to the larger urban population and longer study duration as compared to our study. Longer studies can capture more cases over time, providing a more comprehensive prevalence rate.

In this study, bacterial vaginal infection mixed with vulvovaginal candidiasis (BV/VVC) was the most prevalent form of mixed vaginitis, found in 19.2% of participants. This was followed by TV and BV, which was present in 4.8% of participants. Our findings align with those of Wang et al. in China, where over 15% of women with mixed vaginal infections had BV/VVC as the most common type identified [22]. On the other hand, Bignoumba et al. in Gabon observed that aerobic vaginitis (AV) mixed with BV was the most prevalent form of mixed vaginal infection among reproductive-age females attending gynecologic clinics [23]. This higher prevalence of AV mixed infections can be attributed to the inclusion of aerobic vaginal bacteria in their study. Similar results were reported by Jahic et al. in Bosnia, where anaerobic vaginal bacteria mixed with VVC were the most prevalent, accounting for 30% of women with mixed vaginal infections [24]. The difference in findings may be due to variations in the study populations and the specific focus on aerobic bacteria in the study by Jahic and colleagues. Fan et al. found that AV mixed infections were common in their study in Tianjin, China, with AV mixed with VVC present in 38.1% of cases, AV mixed with BV in 36.9%, and AV mixed with TV in 25% of cases [25]. This highlights the diverse patterns of mixed vaginitis and the importance of considering multiple pathogens in diagnosis and treatment. Sobel et al. noted that mixed vaginitis often requires specific therapy for complete eradication of concurrent manifestations [1].

Socioeconomic factors, healthcare access, and regional hygiene practices also influence the prevalence and patterns of mixed infections. In this study women residing in rural areas were significantly more likely to have mixed vaginal infections compared to those living in urban areas (aOR = 2.9, 95% CI: 1.1–7.5, *p* = 0.03). This finding is consistent with the study by Shawaky et al. in Egypt, which also found higher rates of mixed infections in rural populations due to limited access to healthcare and poor hygiene practices [6]. Similarly, Li et al. in China reported higher infection rates in rural women, attributing it to lower health literacy and socio-economic challenges [26]. However, Rivers et al. in the United States did not find a significant difference between rural and urban populations, possibly due to better overall healthcare access across different regions. [27].

In this study HIV-positive women had a significantly higher likelihood of mixed vaginal infections compared to HIV-negative women (aOR = 4.5, 95% CI: 1.4–14.3, *p* = 0.01). This is supported by findings from Mulu et al. in Ethiopia, where HIV-positive women showed higher rates of mixed infections due to their compromised immune systems [28]. Similarly, a study by Donders et al. in China also indicated higher susceptibility among HIV-positive women. The immune suppression caused by HIV facilitates the proliferation of multiple pathogens, making these women more prone to mixed infections [29].

Women with two or more previous vaginal infections had a significantly higher risk of mixed infections compared to those without (aOR = 9.5, 95% CI: 2.2–41.1, *p* = 0.001). This finding is consistent with the study by Alawkally et al. in Libya, which reported that recurrent infections disrupt the vaginal flora, making women more susceptible to mixed infections [30]. Similarly, Sobel et al. highlighted that recurrent vaginitis often leads to a complex microbial environment (by disruption of normal flora), hence favoring mixed infections [1].

Women with two or more sexual partners were significantly more likely to have mixed vaginal infections compared to those with only one partner (aOR = 5.5, 95% CI: 1.3–23.8, *p* = 0.02). This is in line with findings in Uganda, where multiple sexual partners were associated with higher rates of mixed infections due to increased exposure to different pathogens [31]. However, a study in Turkey did not find a significant association, possibly due to differences in sexual behavior and cultural factors [32].

Women who practiced vaginal douching had a significantly higher risk of mixed vaginal infections compared to those who did not (aOR = 4.6, 95% CI: 1.6–13.3, *p* < 0.001). This finding is supported by research from Fan et al. in China, which demonstrated that douching disrupts the vaginal microbiome, making it more susceptible to infections [25]. Similarly, a study by Wireko et al. in Ghana highlighted the negative impact of douching on vaginal health [33].

### 4.1. Study Strengths and Limitations

This is the first documented research on Mixed Vaginal Infections and their Predictors Among Women with Abnormal Vaginal Discharges in Uganda, particularly at FRRH. The study was conducted in a single tertiary-level hospital, which may limit generalizability to primary and private facilities within the region.

## 5. Conclusions

The study found a high prevalence of mixed vaginal infections, aligning with findings in Egypt and China, underscoring the need for consistent diagnostic protocols and tailored public health strategies. The most common mixed infection was BV/VVC, followed by TV/BV, highlighting the need for comprehensive diagnostic and therapeutic approaches.

Key factors associated with mixed vaginal infections included rural residence, HIV-positive status, a history of multiple vaginal infections, multiple sexual partners, and vaginal douching, highlighting the importance of targeted prevention and management strategies.

## 6. Recommendations

We recommend routine screening for mixed vaginal infections in women with abnormal vaginal discharges, especially in high-prevalence areas. Furthermore, there is a need to educate women, particularly in rural areas, on risks such as multiple sexual partners, HIV, and vaginal douching, and promote specialized care for HIV-positive individuals to reduce mixed infection rates. Develop mobile clinics and community health programs to improve access to sexual health services and education on safe sexual practices and hygiene.

## Figures and Tables

**Table 1:** Sociodemographic characteristics of participants (*N* = 146).

Variables	Categories	Frequency	Percentages (%)
Age	< 20	11	7.5
	20–29	51	34.9
	30–39	51	34.9
	≥ 40	33	22.6

Residence	Rural	69	47.3
	Urban	77	52.7

Marital status	Married	83	56.9
	Single	63	43.2

Level of education	No formal education	22	15.1
	Primary	52	35.6
	Secondary	45	30.8
	Tertiary	27	18.5

Occupation	Formal employee	8	5.5
	Informal employee	48	32.9
	Unemployed	90	61.6

**Table 2:** Factors associated with mixed vaginal infections among women with abnormal vaginal discharges attending gynecological clinic at FRRH (*N* = 146).

Variable	Category	Mixed vaginal infections		cOR (95% CI)	*p* value	aOR (95% CI)	*p* value
		No	Yes				
Age	< 20	5 (45.5)	6 (54.5)	2.8 (0.7–11.2)	0.16^∗^	2.1 (0.4–11.3)	0.41
	20–29	39 (76.5)	12 (23.5)	0.7 (0.3–1.9)	0.49	1.4 (0.5–4.4)	0.55
	30–39	38 (74.5)	13 (25.5)	0.8 (0.3–2.1)	0.63	1.4 (0.3–5.6)	0.66
	≥ 40	23 (69.7)	10 (30.3)	Ref		Ref	

Residence	Urban	43 (62.3)	26 (37.7)	Ref		Ref	
	Rural	62 (80.5)	16 (19.5)	2.5 (1.2–5.2)	0.02^∗^	2.9 (1.1–7.5)	0.03^∗∗^

Marital status	Married	62 (74.7)	21 (25.3)	Ref			
	Single	43 (68.3)	20 (31.7)	1.4 (0.7–2.8)	0.39		

Level of education	No formal education	13 (59.1)	9 (40.9)	1.6 (0.5–5.4)	0.41		
	Primary	38 (73.1)	14 (26.9)	0.9 (0.3–2.4)	0.8		
	Secondary	35 (77.8)	10 (22.2)	0.7 (0.2–2.0)	0.48		
	Tertiary	19 (70.4)	8 (29.6)	Ref			

Occupation	Formal employee	6 (75.0)	2 (25.0)	Ref			
	Informal employee	39 (81.3)	9 (18.7)	0.7 (0.1–4.0)	0.68		
	Unemployed	60 (66.7)	30 (33.3)	1.5 (0.3–7.9)	0.63		

Cigarette smoking	No	100 (73.5)	36 (26.5)	Ref			
	Yes	5 (50.0)	5 (50.0)	2.8 (0.8–10.2)	0.12^∗^	3.4 (0.5–21.3)	0.2

HIV status	No	93 (75.6)	30 (24.4)	Ref		Ref	
	Yes	12 (52.2)	11 (47.8)	2.8 (1.1–7.1)	0.03^∗^	4.5 (1.4–14.3)	0.01^∗∗^

Number of previous vaginal infections in the past 12 months	0	30 (83.3)	6 (16.7)	Ref		Ref	
	1	57 (75.0)	19 (25.0)	1.7 (0.6–4.6)	0.33	2.3 (0.6–8.0)	0.21
	≥ 2	18 (52.9)	16 (47.1)	4.4 (1.5–13.4)	0.01^∗^	9.5 (2.2–41.1)	0.001^∗∗^

Number of sexual partners	1	100 (76.3)	31 (23.7)	Ref		Ref	
	≥ 2	5 (33.3)	10 (66.7)	6.5 (2.1–20.3)	< 0.01^∗^	5.5 (1.3–23.8)	0.02^∗∗^

Vaginal douching	No	56 (64.4)	31 (35.6)	Ref		Ref	
	Yes	49 (83.1)	10 (16.9)	2.7 (1.2–6.1)	0.02^∗^	4.6 (1.6–13.3)	< 0.001^∗∗^

History of abortions	No	28 (60.9)	18 (39.1)	Ref		Ref	
	Yes	77 (77.0)	23 (23.0)	2.2 (1.0–4.5)	0.05^∗^	2 (0.8–5.5)	0.16

Menopause	No	99 (73.3)	36 (26.7)	Ref		Ref	
	Yes	6 (54.6)	5 (45.4)	2.3 (0.7–8.0)	0.19^∗^	3.5 (0.6–21.2)	0.17

*Note:p* ^∗∗^ ≤ 0.05 and *p*^∗^: ≤ 0.2.

Abbreviations: CI, confidence Interval; cOR, crude odds ratio.

## Data Availability

The dataset used in this study is available upon request from the corresponding author, Theoneste Hakizimana: Email: theonestehakizimana5@gmail.com.

## Nomenclature

BV: Bacterial vaginosis
VVC: Vulvovaginal candidiasis
TV: *Trichomonas vaginalis*
FRRH: Fort Portal Regional Referral Hospital
aOR: Adjusted odds ratio
CI: Confidence interval
SDGs: Sustainable Development Goals
SOPs: Standard Operating Procedures
SPSS: Statistical Package for the Social Sciences
NAATs: Nucleic acid amplification tests
AV: Aerobic vaginitis
BSU-REC: Bishop Stuart University–Research and Ethics Committee
HIV: Human immunodeficiency virus
KIU: Kampala International University
